# Fatal Retroperitoneal Hematoma in a Patient Receiving Enoxaparin for Bilateral Pulmonary Emboli

**DOI:** 10.1155/2020/4805967

**Published:** 2020-05-29

**Authors:** Jordan Sexe, Robin McCarthy, Navid Dara, Lyon Brown, Gaurav Dutta

**Affiliations:** ^1^William Carey University College of Osteopathic Medicine, Hattiesburg, MS, USA; ^2^Internal Medicine Residency Program, Baptist Memorial Hospital-Golden Triangle, Columbus, MS, USA; ^3^Department of Pulmonary and Critical Care, Baptist Memorial Hospital-Golden Triangle, Columbus, MS, USA

## Abstract

Venous thromboembolism occurs when a deep vein thrombosis travels to the lungs and forms a pulmonary embolism. Low-molecular-weight heparins are a mainstay in the treatment and prevention of venous thromboembolism and should be initiated promptly due to substantial morbidity and mortality. A rare side effect of low-molecular-weight heparins is major bleeding, which also carries a significant morbidity and mortality rate. Here, we present a case of a fatal retroperitoneal hematoma in a patient being treated with enoxaparin for bilateral pulmonary emboli.

## 1. Introduction

A venous thromboembolism (VTE) occurs when a deep vein thrombosis (DVT) migrates to a pulmonary artery, forming a pulmonary embolism (PE). A subsegmental pulmonary embolism (SSPE) involves subsegmental pulmonary artery branches and spares major vessels. Enoxaparin is a low-molecular-weight heparin (LMWH) commonly used in both the treatment and prevention of PE. LMWHs are a highly effective and popular treatment option due to lack of required laboratory monitoring, low cost, and ease of subcutaneous dosing [1]. For these reasons, LMWHs are a preferred initial agent in the treatment of PE in the acute setting. Direct oral anticoagulants (DOACs), such as rivaroxaban and apixaban, are another popular treatment for PE due to their fast onset of action, affordability, and oral administration. DOACs are a preferred treatment for stable PE in the outpatient setting but are not indicated for treating unstable PE. Unfractionated heparin is the preferred treatment for hemodynamically unstable PE due to its shorter half-life and presence of a well-established reversibility agent, protamine sulfate [1].

A potential consequence of enoxaparin therapy is major bleeding, which has been reported to occur in about 3.79 per 1,000 patients [2]. Major bleeding has been defined as requiring transfusion, causing symptoms in an essential organ/body region, necessitating hospitalization, or fatal [2]. Though very rare, case reports exist of rectus sheath and retroperitoneal hematoma development secondary to enoxaparin administration. Here, we present a patient receiving therapeutic enoxaparin who developed a rectus sheath hematoma and subsequent fatal retroperitoneal hematoma.

## 2. Case Presentation

A 50-year-old female presented to the ED with the complaint of near syncope for the past week. Her past medical history included anxiety, hypertension, migraines, depression, and gastric bypass 10 years priorly. Her home medications were amitriptyline, clonazepam, erenumab, metoprolol, mirtazapine, montelukast, myrbetriq, pantoprazole, ropinirole, and estradiol, which had been started by the patient's PCP one month priorly. The review of systems was positive for nausea and lightheadedness.

Pertinent physical exam findings were hypotension and moderate obesity, with a weight of 146.6 kg. Urinalysis revealed positive nitrites and numerous bacteria. Initial lactic acid was 2.8 mmol/L. Hemoglobin was 12.7 g/dL and hematocrit 40%. The serum creatinine was 1.3 mg/dL. A CT chest/abdomen/pelvis revealed bilateral subsegmental pulmonary emboli. Ultrasound of the lower extremities demonstrated a right-sided DVT of the femoral and popliteal veins. A transthoracic echocardiogram (TTE) showed no evidence of right heart strain, no right atrial dilation, a left ventricle ejection fraction of 55–60%, and left ventricular hypertrophy. An arterial blood gas (ABG) was performed and is shown in Table 1. The HAS-BLED score was 1, indicating a low risk for major bleeding. The aPTT was 26.4 seconds. She was started on therapeutic subcutaneous enoxaparin injections at a dose of 1 mg/kg, totaling 160 mg twice daily. Despite fluid resuscitation, the patient remained hypotensive and was admitted to the ICU for vasopressor support, anticoagulation, and intravenous antibiotics.

**Figure 1:**
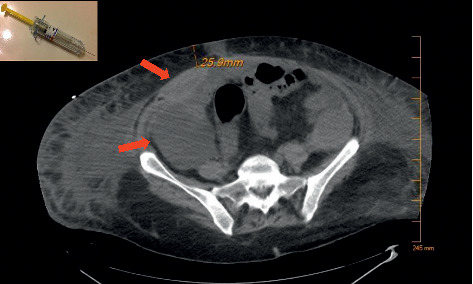
CT performed on day 8 showing right-sided rectus sheath and pelvic hematomas. The inferior epigastric artery was measured approximately 25.9 mm below the skin. A standard enoxaparin injection needle at our facility has a length of 15.9 mm. Photo of the needle was adapted from https://tinylittlehuman.wordpress.com/2012/11/02/lovenox/.

Over the next 48 to 72 hours, the patient improved and was moved to the general medicine floor. However, on day 8, the patient developed sudden, profound hypotension requiring dual pressor support. CBC revealed a hemoglobin of 5.2 g/dL and hematocrit of 16.7%. An ABG revealed a pH of 6.95 and lactate of 14.3 mmol/L (Table 1). Repeat CT imaging revealed a right-sided rectus sheath, and pelvic hematoma presumed to be of inferior epigastric artery origin (Figure 1). A coil embolization was performed with successful ligation of the vessel.

**Figure 2:**
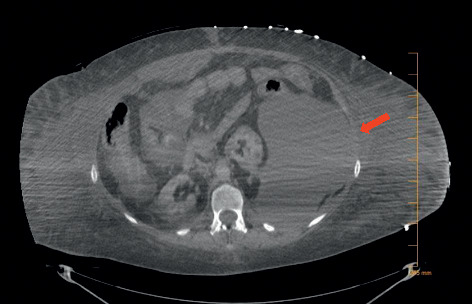
CT performed on day 9 showing development of a massive left-sided retroperitoneal hematoma.

Over the next 24 hours, the patient remained hemodynamically unstable despite dual pressor support requiring repeat imaging, which revealed a new left retroperitoneal hematoma (Figure 2). CT imaging and diagnostic angiography were unable to find a source of the retroperitoneal hematoma. Due to severe hemodynamic instability and an indeterminate source of bleeding, the patient was deemed a poor surgical candidate. Over the next 3 days, the patient required triple pressor therapy and mass transfusion protocol for worsening hemorrhagic shock. Attempts at reversal of anticoagulation with protamine, vitamin K, desmopressin, and tranexamic acid were unsuccessful. The patient died 2 days later.

## 3. Discussion

Enoxaparin is a LMWH commonly used in the treatment and prevention of VTE. A meta-analysis found that LMWH had a lower rate of major hemorrhage compared to intravenous heparin and subcutaneous unfractionated heparin during initial VTE treatment [3]. Bleeding is considered major when it is located within the retroperitoneum or cranium, or if it leads to transfusion, hospitalization, or death [4]. Retroperitoneal hemorrhage constitutes 1% of all adverse bleeding events [4]. Though more common in patients with renal impairment, the rarity of rectus sheath and retroperitoneal hematomas makes well-established conclusions difficult [5, 6].

Diagnosis is commonly confirmed with CT imaging [7]. Treatment consists of fluid resuscitation, blood transfusions, FFP, and discontinuing anticoagulation. Administration of protamine has questionable benefit with a 40–70% effectiveness in reversing LMWH [8]. If conservative therapy fails, surgical intervention may be explored if a bleeding vessel is identified [7].

Here, it is believed that an enoxaparin injection pierced the patient's inferior epigastric artery. There have been numerous reports of rectus sheath hematoma development secondary to accidental injection of intramuscular tissue or the inferior epigastric artery with enoxaparin [9, 10]. This patient's rectus sheath hematoma was not present on initial CT imaging performed in the ED but became apparent on CT imaging 8 days after initiation of enoxaparin. This vessel was fortunately able to be identified and successfully coil embolized. However, the ultimate fatal retroperitoneal hematoma was likely secondary to cannulation of the femoral artery during embolization of the rectus sheath hematoma in a partially heparinized patient.

It is also possible that discontinuing the patient's anticoagulation provoked clinical deterioration. The enoxaparin was discontinued after the patient developed major bleeding in the presence of acute hypotension on day 8. However, it could be possible that stopping anticoagulation in a patient with bilateral subsegmental pulmonary emboli while immobilized during hospitalization may have worsened the underlying pulmonary emboli and/or elicited formation of a new PE from one of the patient's proximal DVTs. Regardless, this patient developed a massive retroperitoneal hematoma and ultimately died of complications from hemorrhagic shock.

Proper administration of subcutaneous enoxaparin injections relies on pinching a segment of subcutaneous tissue and injecting the needle into the isolated area. Some practitioners may administer the injection by simply pressing into the anterior abdominal wall without isolating a secure subcutaneous fat pad. Such a technique could compress the superficial fat and lead to injection of intramuscular tissue or a blood vessel. In Figure 1, we show the depth of this patient's inferior epigastric artery in relation to the length of needle typically used in enoxaparin injections. This shows the plausibility of a routine enoxaparin injection piercing the inferior epigastric artery if not administered correctly.

## 4. Conclusion

It is imperative to closely monitor for hemodynamic instability and symptomatic changes related to potential hemorrhaging in patients receiving enoxaparin. In addition, proper training of medical staff with regard to administration is of the utmost importance in preventing complications. Though rare, LMWHs such as enoxaparin may precipitate adverse hematologic events that may contribute to morbidity and mortality.

## Figures and Tables

**Table 1:** The patient's ABG results.

	pH	pO_2_ (mmHg)	pCO_2_ (mmHg)	HCO_3_ (mmol/L)	O_2_ saturation
Day 1	7.45	93	28 (L)	19.5 (L)	98
Day 8	6.95	361 (H)	18 (L)	4.0 (L)	98
Day 11	7.40	49 (L)	43	26.2	91 (L)

The ABG on day 8 was performed just after the patient developed acute hypotension and demonstrates a severe acidosis. The ABG on day 11 was performed about 3 hours prior to death and demonstrates marked hypoxemia despite high-flow oxygen therapy.
